# Analysis of the role of the interleukins in colon cancer

**DOI:** 10.1186/s40659-020-00287-2

**Published:** 2020-05-07

**Authors:** Xiyi Wei, Yuan Zhang, Zhou Yang, Yeqin Sha, Yitong Pan, Yusheng Chen, Lei Cai

**Affiliations:** 1 Department of General Surgery, Shanghai Pudong Hospita, Fudan University Pudong Medical Center, 2800 Gongwei Road, Huinan Town, Pudong, 201399 Shanghai, China; 2 First Clinical Medical College of Nanjing Medical University, 210029 Nanjing, China; 3 Department of Bioinformatics, School of Biomedical Engineering and Informatics, Nanjing Medical University, 211116 Nanjing, China

**Keywords:** Colon cancer, Interleukins, Tumor progression

## Abstract

**Background:**

The role of interleukin family in colon cancer remained controversial. The purpose of this study was to investigate the association between interleukin family and colon cancer progression through bioinformatics methods and to validate such association in clinical patients.

**Methods:**

A total of 15 differentially expressed interleukins between the colon cancer tissue and normal colon tissue were evaluated from the Cancer Genome Atlas (TCGA) database with R software and only interleukin-7 (IL-7) was significantly associated with survival. The signaling pathway associated with IL-7 was then investigated using gene enrichment analysis. In addition, subsets of TNM were analyzed in detail and univariate and multivariate COX regression analysis were conducted. Finally, we performed western blotting, immunohistochemistry, cell proliferation and cell apoptosis analysis to examine the expression of IL-7 in patients with intestinal cancer.

**Results:**

The study demonstrated that IL-7 could inhibit the progression of colon cancer. In addition, IL-7 was found to be associated with overall survival (OS) and pathological stage. Further analysis of IL-7 expression with clinical data indicated that IL-7 was a key factor in inhibiting colon cancer progression.

**Conclusion:**

IL-7 was a key factor in inhibiting the progression of colon cancer and was closely related to overall survival.

## Background

Colon cancer, a major malignancy of the alimentary canal, ranked third among malignant tumors in terms of morbidity worldwide [1, 2]. Relevant study revealed that more than 1 million people developed colon cancer each year, and the disease-specific mortality rate in developed countries was approximately 33% [3]. Mortality of colon cancer are on the rise due to changes in diet and lifestyle [4, 5]. Although colon cancer treatment options (e.g. surgery, chemoradiotherapy, and immunotherapy) have been greatly improved, the 5-year survival rate remained as high as 50% [6].

Interleukins, a class of cytokines, were first discovered in the 1970s as a broader cellular messenger molecule family that allowed cells of the immune system to communicate with one another and produce coordinated, as well as specific responses to target antigens [7, 8]. In addition, interleukins had a variety of immunomodulatory functions that direct the maturation, differentiation, migration and adhesion of immune system cells [9, 10]. Previous studies have found that interleukins can regulate tumor microenvironment and participate in tumor development and progression [11–13]. However, the role of the entire interleukin family in colon cancer remained controversial. Therefore, it was necessary to probe the role of the interleukin family in the colon cancer micro-environment.

The Cancer Genome Atlas (TCGA) was regarded as the largest cancer database, containing samples of more than 20,000 primary cancers and normal matched samples of multiple cancer types. Therefore, we can investigate tumor gene data in greater depth with bioinformatics methods. Further, it can be linked to clinical data in order to obtain more valuable and meaningful results [14]. To assess the association between the interleukin family and progression of colon cancer, our study analyzed the mRNA expression of colon adenocarcinoma in TCGA by R software and validated it in clinical patients.

## Materials and methods

### TCGA data acquisition and differentially expressed interleukin gene analysis

The colon adenocarcinoma data in TCGA contained 480 colon cancer cases and 41 paracancerous control cases with basic information, including age, gender, tumor pathologic stage, and Tumor & Lymph Node & Metastasis (TNM) stage (Table 1). All mRNA expression data were downloaded and further analyzed by R software along with clinical data.

**Table 1:** Clinical characteristics of patients

T stage	
T1	8
T2	66
T3	272
T4	46
M stage	
M0	330
M1	62
N stage	
N0	235
N1	89
N2	68
Age	
≤ 65	152
> 65	240
Gender	
Female	186
Male	206
Stage	
Stage I	67
Stage II	160
Stage III	103
Stage IV	62

We used the R software “Limma” package to normalize the original expression levels of mRNAs downloaded from TCGA. “Limma” package was then further utilized to analyze the expression of each interleukin gene between cancer and normal tissues, setting P < 0.05 as the filter condition for differentially expressed interleukin.

### Gene set enrichment analysis of colon cancer

Gene enrichment analysis (GSEA) (version 3.0, the broad institute of MIT and Harvard, http://software.broadinstitute.org/gsea/downloads.jsp) was conducted between colon cancer and paracancerous normal tissues to study the biological characteristics of colon cancer. In detail, the “collapse data set to gene symbols” was set to false, the number of marks was set to 1000, the “permutation type” was set to phenotype, the “enrichment statistic” was set to weighted, and the Signal2Noise metric was used for ranking genes. High expression group was used as experimental group and low expression group was used as reference group. “c2.cp.kegg.v7.0.symbols.gmt” gene sets database was used for enrichment analysis. Gene set size > 500 and < 15, FDR < 0.25, and nominal *P* value < 0.05 were regarded as the cut-off criteria.

### Overall survival curve and TNM subsets analysis

Based on the TCGA database, OS curves was drawn by the R software through Kaplan–Meier analysis. P < 0.05 was considered to be significant for the impact of OS. The association between each subset of TNM and IL-7 was analyzed by R software based on the TCGA database via the Wilcox test. The 7th edition of the TNM stage system 23 was adopted, and Mx was defined as unable to evaluate the presence or absence of distant metastasis.

### Immunohistochemical (IHC) staining

IHC was performed on paraffin-embedded sections. The sections were deparaffinized in xylene and hydrated with decreasing concentrations of ethanol (100, 90, 80, 75%) for 3 min each time and microwaved-heated in sodium citrate buffer for antigen retrieval. Then, the sections were blocked in 5% BSA and incubated with anti- IL-7 rabbit polyclonal antibody (1:100, R&D Systems, MN, USA) at 4 °C overnight. Next, the sections were treated with horseradish peroxidase (HRP)‑conjugated rabbit secondary antibody (1:200; ProteinTech Group) for 60 min at room temperature; then, 3,3′‑diaminobenzidine development (DAB Substrate Chromogen System; Dako) and hematoxylin staining were performed. The sections were fixed and images were obtained with inverted microscope (Olympus IX71, Japan).

### Cell Lines and regents

The human colon cancer cell line HCT116 and RKO were purchased from the University of Colorado Cancer Center Cell Bank and cultured in RPMI 1640 medium supplemented with 10% FBS (Invitrogen, Carlsbad, CA, USA) at 37 °C in a 5% CO_2_ atmosphere. Cells were digested and passaged when cell fusion reached 80%. Recombinant Human IL-7 Protein (rhIL-7) was purchased from R&D Systems (MN, USA). The working concentration was 100 nM.

### Protein extraction and western blotting analysis

Total protein of the cells in each group was extracted using RIPA extraction reagents with 1% phenylmethanesulfonyl fluoride (PMSF) as well as 1% DL-Dithiothreitol (DTT). The concentration of the lysate protein was detected by a BCA protein assay kit (Beyotime Biotechnology). Equal amounts (20 μg) of protein, as determined with BCA protein assay kit (Thermo Fisher Scientific, USA) were separated by 10% SDS-PAGE. The proteins were then transferred to PVDF membranes (0.45 mm; Beijing Solarbio Science & Technology Co., China). The membranes were blocked with 5% BSA for 1 h at room temperature and then incubated with IL-7 rabbit polyclonal antibody (1:1000, R&D Systems, MN, USA) antibodies at 4 °C for 12 h. GAPDH rabbit polyclonal antibodies (1:4000, Proteintech, USA) were used as loading controls and normalization. The secondary antibody anti-rabbit antibodies conjugated to HRP (1:4000; ProteinTech Group) were incubated for approximately 1 h at room temperature. Finally, the bands were visualized with ECL reagents (Thermo Fisher Scientific) and Omega Lum G machine (Aplegen, USA).

### Flow cytometry

For cell apoptosis assay, 2 × 10^5^ cells were harvested and washed with PBS for 3 times. Then the samples were resuspended in 100 µl of binding buffer, stained with 5 µl of Annexin–V and propidium iodide (PI), and stored at room temperature for 20 min in the dark. Subsequent to staining, additional 400 µl binding buffer was added in sample and resuspended. Analysis were performed with flow cytometry (Becton–Dickinson, Bedford, MA, USA).

### Cell proliferation assay

3 × 10^3^ cells suspended in 100 µl RPMI-1640 medium were seed into 96-well plate. The cell proliferation was assessed by the CCK8 (Dojindo Molecular Technologies, Japan). 10 µl CCK8 solution was given to each well of the plate after different incubation times: 0 h, 24 h, 48 h and 72 h. Finally, we measured the absorbance at 450 nm wavelength after 2 h incubation.

### Cell invasion assay

Cell invasion was analyzed with transwell plates (24-well insert, 8 μm pore size; BD Biosciences, Bedford, MA, USA). The filters (Corning Inc., USA) were coated with 55 μL Matrigel (1:8 dilution; BD Biosciences). The 10^4^ cells were suspended in 100 μl RPMI-1640 medium without serum and seeded in the upper chamber. Next, 600 μl 90% RPMI-1640 supplement with 10% FBS was added to the bottom chamber. After incubation for 24 h, the chambers were fixed by 4% paraformaldehyde for 30 min and then stained by 0.1% crystal violet for 30 min. At last, we used a magnification microscope to count the amount of the invasion cells in the bottom of the chamber.

### Statistical analysis

The experiments in this article were performed in triplicate, and the data were expressed as mean ± standard deviation. A t-test was utilized for the statistical analysis of the data from 2 groups. The comparisons of multiple groups were performed with one-way ANOVA followed by an LSD-t test. P < 0.05 was considered to be significant.

## Results

### Differentially expressed interleukin genes screening and OS analysis

**Fig. 1:**
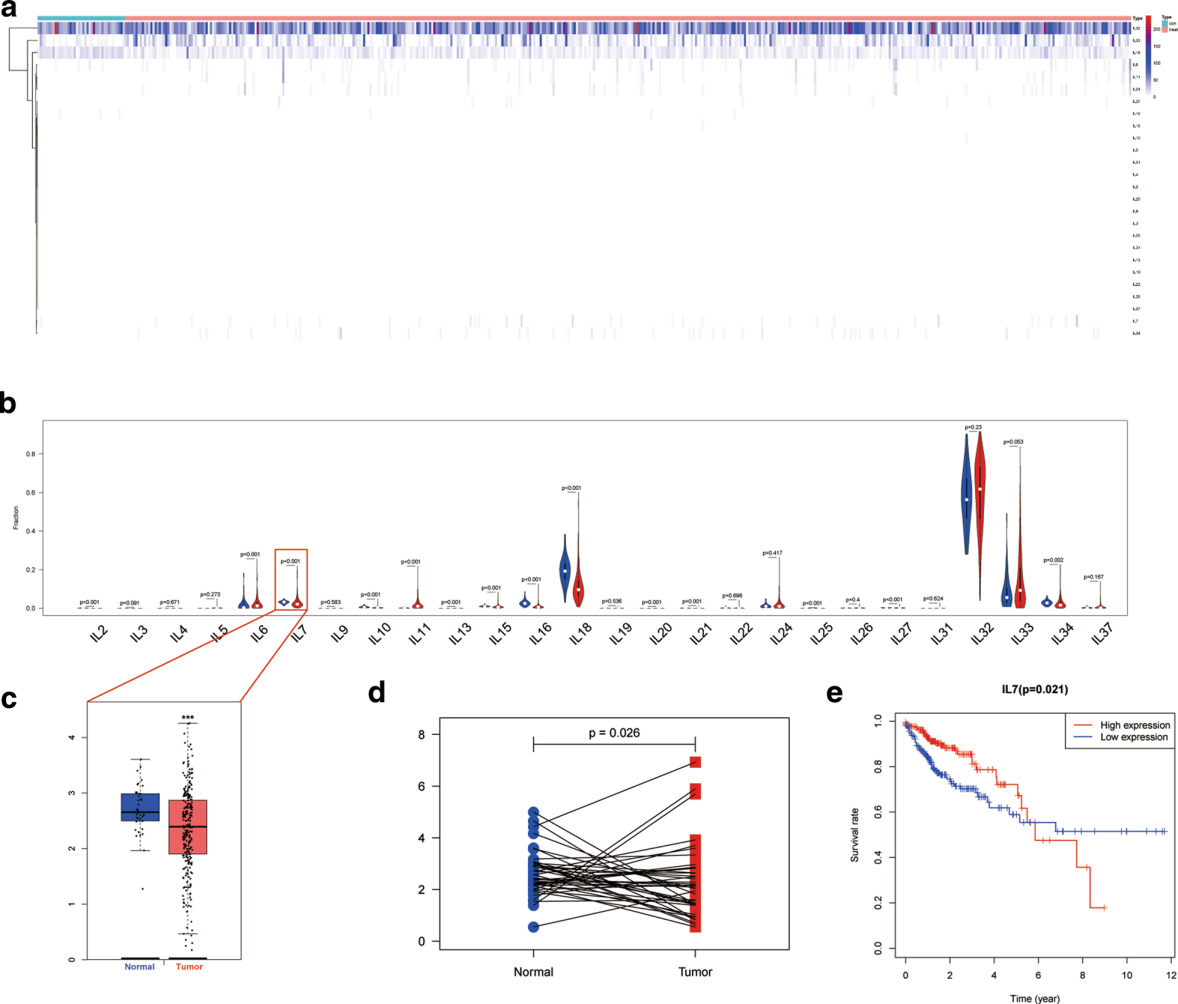
Analysis of the expression level of interleukin family and its survival correlation in 480 colon cancer cases and 41 paracarcinoma case. **a** Heatmap of the expression level of interleukin family in 480 colon cancer cases and 41 paracarcinoma cases. **b** Difference analysis of 26 interleukins between 480 colon cancer cases and 41 paracarcinoma cases. **c** Difference analysis of IL-7 between colon cancer and paracarcinoma group. **d** Paired difference analysis of IL-7 between colon cancer and paracarcinoma group. **e** Correlation analysis between IL-7 expression level and overall survival in 480 colon cancer cases

The expression level of the interleukin family genes was shown in Fig. 1a. R software was utilized to screen differentially expressed interleukin gene extracted from TCGA-COAD. In total, 15 interleukins were differentially expressed between colon cancer and paracancerous tissues, 11 interleukins showed no difference in expression, and 12 were almost non-expressed (Fig. 1b, Table 2). All differentially expressed interleukins were correlated with clinical data of TCGA-COAD for further analyzing OS via R software. We finally found that interleukin 7 was significantly down-regulated in colon cancer compared with normal tissue (Fig. 1c, d). Meanwhile, its down-regulation was significantly associated with worse overall survival (Fig. 1e).

**Table 2:** Screening of differentially expressed interleukin genes between cancer and normal tissue groups

Significant expressed	IL-2, IL-6, IL-7, IL-10, IL-11, IL-13, IL-15, IL-16, IL-18, IL-20, IL-21, IL-25, IL-27, IL-33, IL-34
Insignificant expressed	IL-3, IL-4, IL-5, IL-9, IL-19, IL-22, IL-24, IL-26, IL-31, IL-32, IL-37
Non-expressed	IL-1, IL-8, IL-12, IL-14, IL-17, IL-23, IL-28, IL-29, IL-30, IL-35, IL-36, IL-384

### Gene set enrichment analysis of IL-7

**Fig. 2:**
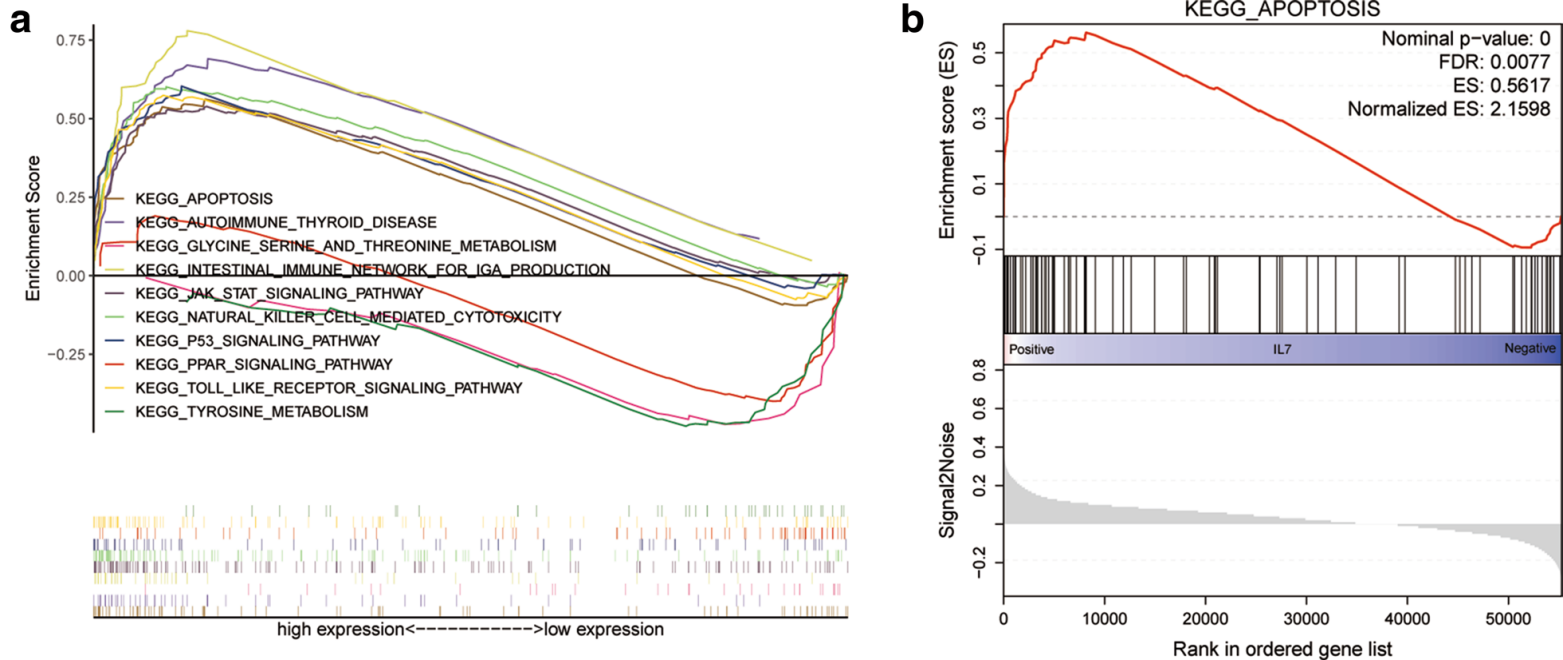
**a** Gene set enrichment analysis of IL-7 in colon cancer. **b** Presentation of apoptotic pathway mediated by IL-7 in gene set enrichment analysis

To explore how IL-7 was involved in colon cancer progression, we performed a GSEA based on the TCGA colon cancer cohort. A total of 100 significant genes were obtained from GSEA and genes with positive correlations were mapped. Further, we found the most important pathways involved in IL-7, including intestinal immune network for IgA production, apoptosis, natural killer, Autoimmune, JAK, P53 signaling pathway, a series of pathways associated with the development of colon cancer. The details were illustrated in Fig. 2a, b.

### TNM subsets analysis

**Fig. 3:**
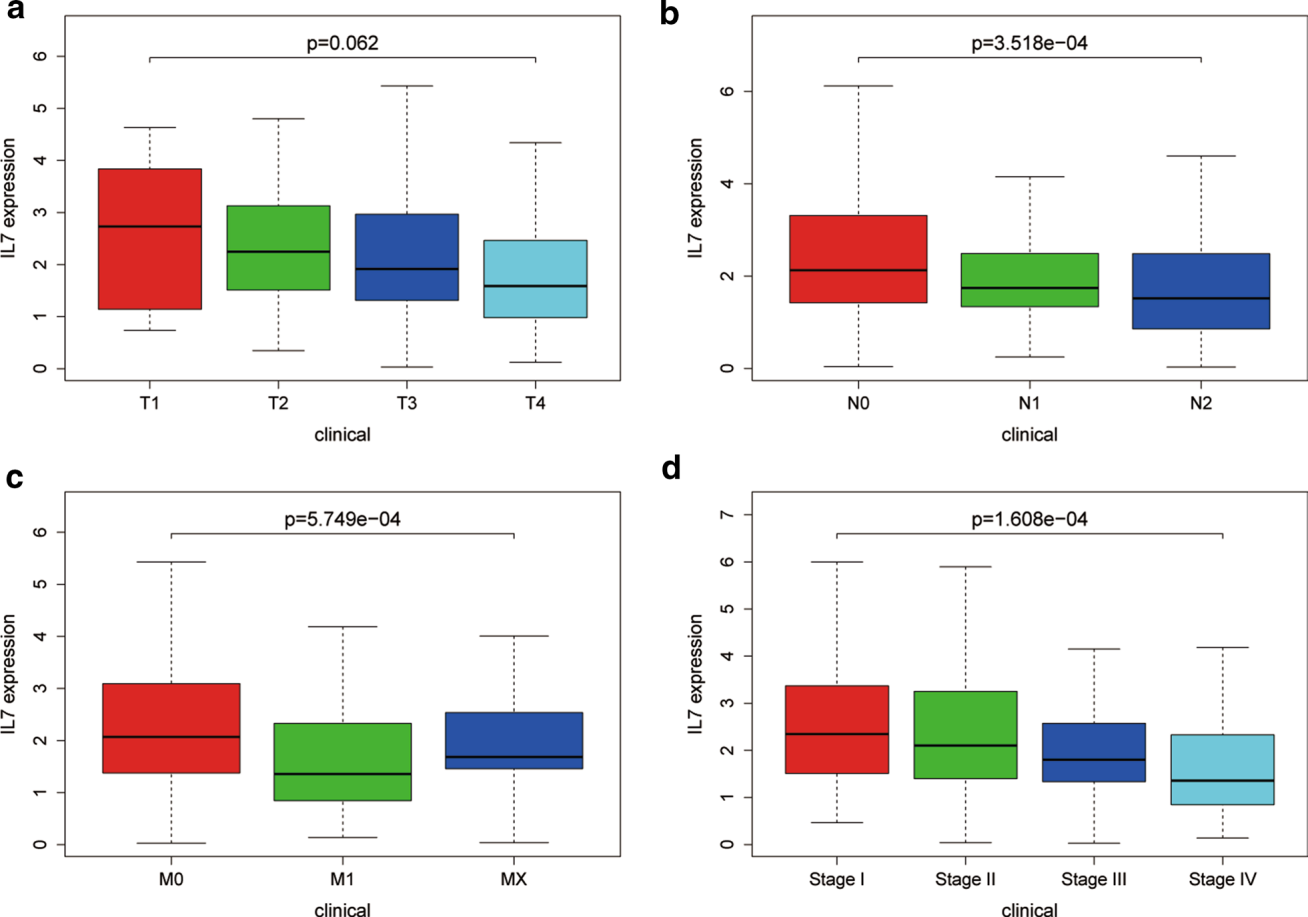
Correlation analysis between TNM&Stage and IL-7 in 480 colon cancer cases. **a** Correlation analysis between tumor stage and IL-7 expression in 480 colon cancer cases. **b** Correlation analysis between node stage and IL-7 expression in 480 colon cancer cases. **c** Correlation analysis between metastasis stage and IL-7 expression in 480 colon cancer cases. **d** Correlation analysis between pathologic stage and IL-7 expression in 480 colon cancer cases

The association between each subset of TNM and IL-7 was analyzed by R software via the Wilcox test. P < 0.05 was considered to be statistically significant. We found that the expression level of IL-7 continuously stepwise decreased in each subgroup of TNM, except for the MX group. In the tumor stage, IL-7 gradually decreased in the subgroup but there was no statistical difference (Fig. 3a). Conversely, we found that IL-7 decreased significantly between subgroups during the node stage (Fig. 3b). In the metastatic stage, IL-7 increased in the Mx phase compared to the M1 phase, but overall IL-7 was statistically different in the metastatic stage (Fig. 3c). In the pathological stage, IL-7 expression gradually decreased and was statistically different (Fig. 3d).

### Univariate and multivariate COX regression analysis

**Fig. 4:**
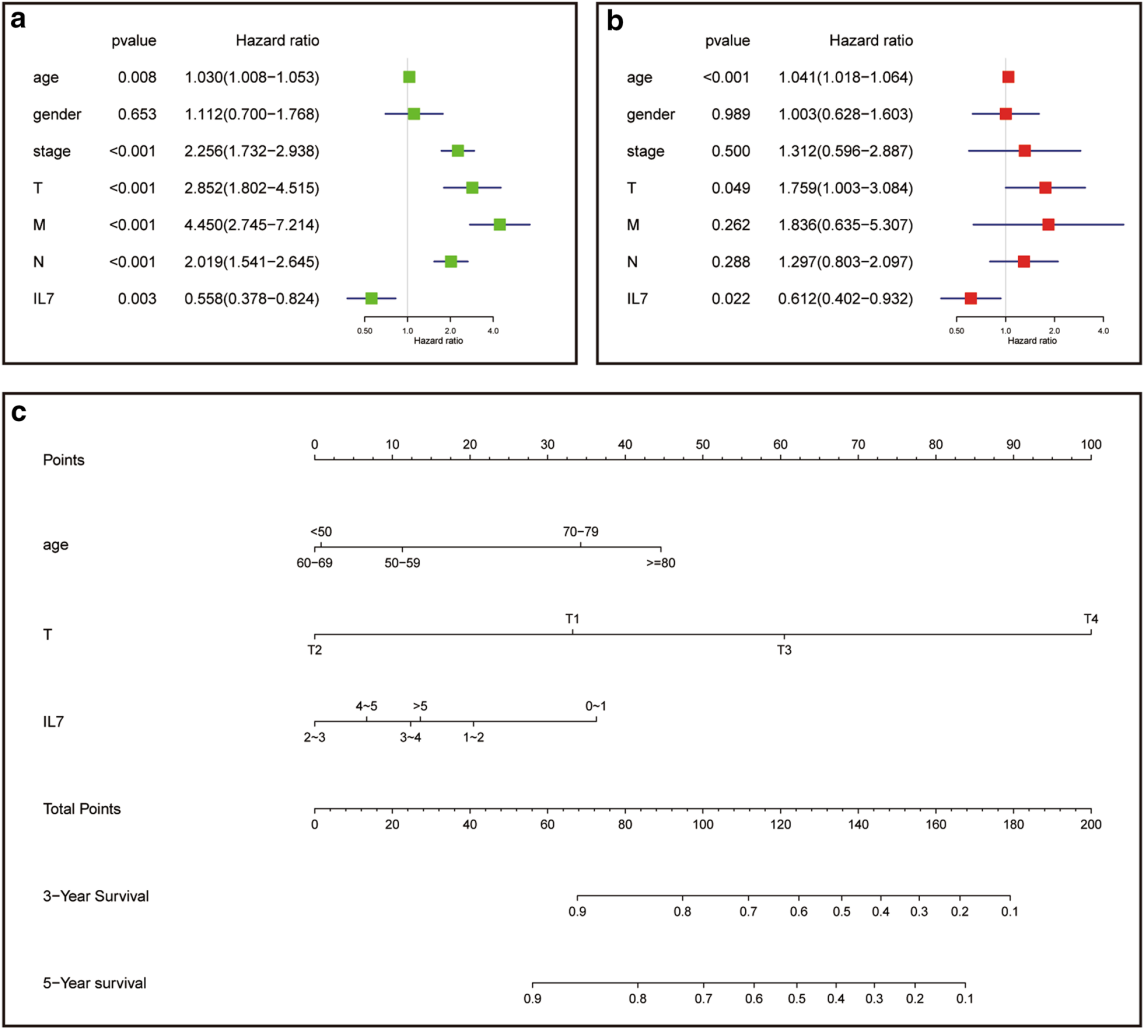
Cox’ s proportional hazard model of correlative factors in colon cancer patients. **a** Univariate COX regression analysis for factors affecting the overall survival. **b** Multivariate COX regression analysis for factors affecting the overall survival. **c** An established nomogram to predict survival based on COX model

Cox’s proportional hazards model was applied to analyze related factors that may affect the overall survival of colon cancer patients. In both univariate and multivariate analysis, high expression of IL-7 suggested better OS. In univariate analysis, the P-value was 0.003, the hazard ratio (HR) was 0.558, and the 95% confidence interval (CI) was 0.377–0.824. In multivariate analysis, the P-value was 0.022, the HR was 0.611, and the 95% CI was 0.401–0.932 (Fig. 4a, b). Further, based on the results of multivariate cox regression analysis, we established a nomogram model that may predict patients’ survival (Fig. 4c).

### IL-7 expression in clinical patients and function in cell line

**Fig. 5:**
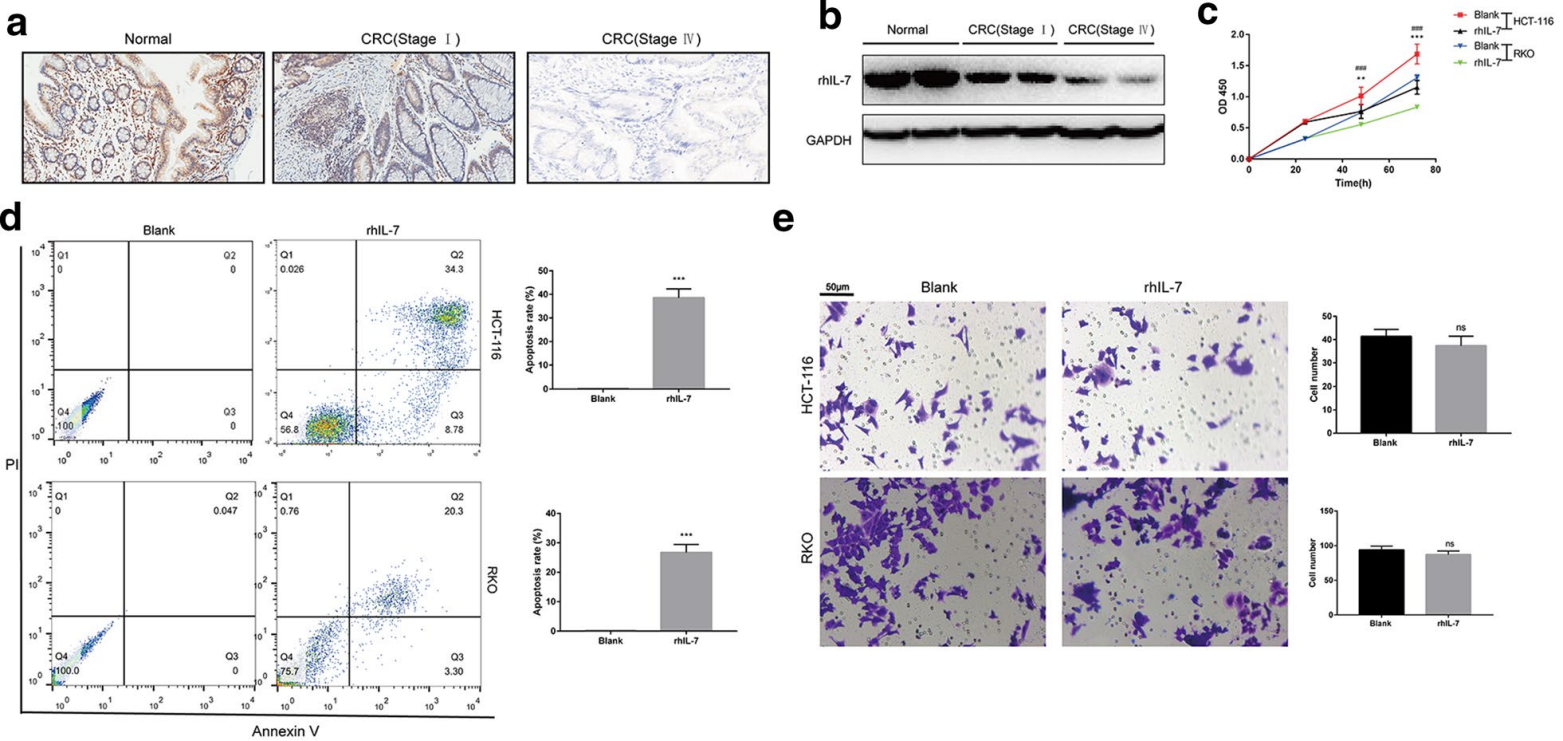
**a** Expression of IL-7 in clinical colon cancer patients (paired normal tissue, pathological stage I and stage IV) performed by IHC (Magnification 400x). **b** Expression of IL-7 in clinical colon cancer patients (paired normal tissue, pathological stage I and stage IV) performed by western blotting. **c** Cell proliferation of HCT116 and RKO (blank *vs* rhIL-7 treatment) detected by CCK8. **d** Cell apoptosis of HCT116 and RKO (blank *vs* rhIL-7 treatment) detected by flow cytometry. **e** Cell invasion of HCT116 and RKO (blank *vs* rhIL-7 treatment) detected by Transwell assay (Magnification 400x). (^***^P<0.001, *ns* no significance)

For further exploration of IL-7 expression in clinical colon cancer patients, Western Blotting and IHC were performed. As expected, IL-7 protein was significantly decreased in early stage colon cancer compared with paired normal tissue, meanwhile the expression of IL-7 in advanced stage colon cancer showed further decrease (Fig. 5a, b). As predicted in GSEA analysis above, we inferred IL-7 promotes apoptosis of colon cancer to repress its progression. In cell proliferation analysis, treatment of rhIL-7 (100 ng/ml, 48 h) significantly decreased the growth of HCT116 and RKO at both 48 and 72 h (Fig. 5c). In cell apoptosis analysis, rhIL-7 significantly increased the apoptosis rate of HCT116 and RKO (Fig. 5d). In addition, we further investigated the effect of rhIL-7 at cell invasion ability, whereas no significant change was found (Fig. 5e).

## Discussion

In the past few years, despite great efforts in colon cancer research, there has been no significant improvement in the 5-year survival rates. Patients with early and unadvanced colon cancer had a better prognosis and can be cured by surgery combined with adjuvant therapy. However, the majority of patients with advanced colon cancer can not undergo a surgical resection alone [15] and the chemotherapy or palliative treatment was the only option for them. Therefore, it was crucial for advanced colon cancer patients to explore more effective prognosis markers and therapeutic targets. To this end, we screened the interleukin family by bioinformatics methods and performed a differential analysis. Interleukins associated with colon cancer progression and survival were further analyzed and eventually validated in clinical patient samples.

In the initial analysis results, 15 interleukins were identified as differentially expressed interleukins between colon cancer and paracancerous tissues. Further analysis confirmed that only IL-7 was associated with cancer progression, accounting for 2.63%. Although the amount of differentially expressed interleukins between the tumor and paracancerous tissue was large, the majority of of them may be meaningless or of little significance, and only a fraction was actually related to the development of the tumor. Therefore, only IL-7 was studied by gene enrichment analysis to evaluate its cellular composition, molecular function and biological characteristics.

In gene set enrichment analysis, Intestinal immune network for IgA production pathway has been shown to be the most relevant pathway for IL-7. Previously, Yang et al. [16] evaluated the function of APOBEC3F gene and intestinal immune network for IgA production pathway in the biological process of hepatocellular carcinoma (HCC). Intestinal immune network for IgA production pathway activated by APOBEC3F may lead to malignant biological behavior of HCC cells by up-regulating pIgR, CCR9, CCR10 and CXCR4 protein levels. This was inconsistent with our results, while intestinal immune pathway played a positive role in inhibiting cancer in our study. Apoptosis was the most important mechanism for inhibiting tumor growth and played an important role in tumorigenesis and immune response. Therefore, we performed a further analysis of the IL-7-mediated apoptosis pathway. Previous studies [17–19] have shown that several biologically active substances could induce tumor cell apoptosis, inhibiting tumor progression by modulating pro-apoptotic proteins and apoptotic proteins (Bax, Bcl-xl) and increasing caspase expression and activation (CASP3, CASP8, CASP9). IL-7 may inhibit the development of colon cancer cells through apoptotic pathway.

We further analyzed the association of IL-7 with survival and TNM. It was found that IL-7 was significantly associated with survival and showed a continuous gradual decline in each subset of TNM. The IL-7 receptor was IL-7R, which, when combined, may results in activation of JAK1 or JAK3, followed by activation of many downstream signaling pathways, including STAT5a/b, PI3 kinase and SRC kinase [20–25]. Further, IL-7 treatment can enhance long-term tumor antigen-specific CD8 + T cell response and greatly prolong the survival of tumor patients [26, 27]. We finally verified the expression of IL-7 in clinical patients and found that IL-7 was significantly down-expressed in colon cancer tissues compared to normal tissues. In the proportional risk model of Cox, high expression of IL-7 indicated a better OS. High expression of IL-7 also showed a negative association with tumor progression in the TNM subgroup analysis.

In another research focused on the role of IL-7 in the progression of colon cancer, the apoptosis of tumor cells treated with combination of OXP and IL-7 was significantly increased, indicating that IL-7 combined with OXP can significantly reduce cell proliferation and induce apoptosis in vivo [28]. Nevertheless, the use of IL-7 alone had no effect on tumor growth in mice, and IL-7 was unlikely to have a direct effect on tumors. Similarly, both of our results confirmed the role of IL-7 in inhibiting tumor progression. However, our results show that IL-7 itself may inhibit the progression of intestinal cancer through the apoptotic pathway.

## Conclusion

In summary, we successfully revealed several significant interleukins for the diagnosis and prognosis of advanced colon cancer. IL-7 was the most meaningful one among them and had been verified in clinical patients. Moreover, the mechanism by which IL-7 inhibited the progression of intestinal cancer required further research.

## Data Availability

The datasets used and analyzed during the current study are available from the corresponding author on reasonable request.

## Abbreviations

TCGA: The Cancer Genome Atlas
IL-7: Interleukin-7
OS: Overall survival
TNM: Tumor & Lymph Node & Metastasis
GSEA: Gene enrichment analysis
rhIL-7: Recombinant human IL-7 protein
HR: Hazard ratio
CI: Confidence interval
